# The Intersection of HIV and Pulmonary Vascular Health: From HIV Evolution to Vascular Cell Types to Disease Mechanisms

**DOI:** 10.3390/jvd3020015

**Published:** 2024-05-06

**Authors:** Amanda K. Garcia, Sharilyn Almodovar

**Affiliations:** 1 Department of Immunology & Molecular Microbiology, Texas Tech University Health Sciences Center, School of Medicine, Lubbock, TX 79430, USA; 2 Center for Tropical Medicine & Infectious Diseases, Texas Tech University Health Sciences Center, School of Medicine, Lubbock, TX 79430, USA

**Keywords:** HIV, endothelial cells, SMC, smooth muscle cells, viral evolution, Nef, gp120, Tat, pulmonary vascular health, NHPs, HIV transgenic models, humanized mice, vasculature

## Abstract

People living with HIV (PLWH) face a growing burden of chronic diseases, owing to the combinations of aging, environmental triggers, lifestyle choices, and virus-induced chronic inflammation. The rising incidence of pulmonary vascular diseases represents a major concern for PLWH. The study of HIV-associated pulmonary vascular complications ideally requires a strong understanding of pulmonary vascular cell biology and HIV pathogenesis at the molecular level for effective applications in infectious diseases and vascular medicine. Active HIV infection and/or HIV proteins disturb the delicate balance between vascular tone and constriction, which is pivotal for maintaining pulmonary vascular health. One of the defining features of HIV is its high genetic diversity owing to several factors including its high mutation rate, recombination between viral strains, immune selective pressures, or even geographical factors. The intrinsic HIV genetic diversity has several important implications for pathogenic outcomes of infection and the overall battle to combat HIV. Challenges in the field present themselves from two sides of the same coin: those imposed by the virus itself and those stemming from the host. The field may be advanced by further developing *in vivo* and in vitro models that are well described for both pulmonary vascular diseases and HIV for mechanistic studies. In essence, the study of HIV-associated pulmonary vascular complications requires a multidisciplinary approach, drawing upon insights from both infectious diseases and vascular medicine. In this review article, we discuss the fundamentals of HIV virology and their impact on pulmonary disease, aiming to enhance the understanding of either area or both simultaneously. Bridging the gap between preclinical research findings and clinical practice is essential for improving patient care. Addressing these knowledge gaps requires interdisciplinary collaborations, innovative research approaches, and dedicated efforts to prioritize HIV-related pulmonary complications on the global research agenda.

## 1. Introduction

In the era of combination antiretroviral therapy (cART), infection with Human Immunodeficiency Virus (HIV) has transitioned into a manageable chronic condition [1]. People living with HIV (PLWH) now have longer life expectancies, owing to the efficacy of cART. However, despite important antiretroviral advancements, several concerns remain regarding the management and prognosis of PLWH. Such challenges include (1) a delayed diagnosis for HIV positivity, as people who do not know their HIV positive status may continue spreading the virus; (2) a lack of screening and timely diagnosis of HIV-associated comorbidities, as the prevalence of such comorbidities may be overlooked and/or disregarded therapeutically; (3) residual HIV replication, which imposes an important barrier to achieving viral suppression and may lead to development of drug-resistant quasispecies; (4) the existence of known (as well as potentially uncharacterized) HIV reservoirs and sanctuaries in the host, which fuels chronic inflammation, leading to end-organ damage while challenging eradication strategies by contributing to the pool of residual replication; (5) limited access to antiretroviral therapy and essential healthcare services in resource-limited settings and marginalized populations; (6) antiretroviral drug toxicities, which prompt antiretroviral treatment interruptions, reduced adherence, and overall compromised prognosis; (7) the compromised ability of some patients to sustain viral suppression; (8) the alarming increase in transmissible drug resistance mutations, which emphasizes the importance of ongoing clinical monitoring of arising viral strains; (9) the complicating scenarios when multiple hits are introduced, such as smoking and the use of recreational and/or illegal drugs, which exacerbate HIV-associated comorbidities; and lastly, (10) the strong evolutive capacity of HIV, which makes it a difficult moving target for research and effective vaccine development.

PLWH face a growing burden of chronic diseases including liver fibrosis, sclerosis, cirrhosis, male and female infertility, bipolar disorders, depression, cervical malignancies, co-infections, diabetes, and lung and cardiovascular diseases [2–7]. The wide spectrum of such HIV-associated comorbidities is due to chronic inflammation. In this review, we will focus on HIV-mediated pulmonary vascular diseases, with a particular emphasis on pulmonary hypertension (PH) as a model for severe pulmonary vascular remodeling. PH started as a clinical observation in HIV-infected individuals, which quickly populated the scientific literature three decades ago, through numerous case reports [8–13]. After almost a decade of observations, HIV was recognized as an independent risk factor for PH, along with commendable efforts using drugs to target either the lungs or the virus to tackle HIV-associated PH [14–17].

It is known that PH is a substantial cause of death in PLWH; however, the exact prevalence of PH in the HIV population is difficult to determine and may be underestimated because of asymptomatic patients. The traditional prevalence of HIV-PH has been reported as 0.5% since the early 1990s, which was before the availability of highly active antiretroviral treatments [18]. Several groups have performed more recent studies and reported higher prevalence when measuring PH in HIV-infected groups. In a recent cross-sectional study published in early 2024, Mahajan et al. determined the prevalence of HIV-PH in a group of 150 HIV-positive patients receiving first-line antiretroviral treatment with no prior clinical history of chronic cardiopulmonary disease. Based on systolic pulmonary artery pressures determined using a two-dimensional M mode echocardiogram, the prevalence of PH was 8% [19]. This is near the detection of PH in 8.3% of HIV-infected adults by Bigna et al. in 2019 and 6.1% of 374 HIV-infected patients found by Schwarze-Zander C et al. in 2015 [20,21]. Another study published in early 2024 by Denu et al. determined the prevalence of PH to be 17.4% in 362 PLWH [22]. This aligns with data from another cross-sectional study published in 2022 by Harimenshi et al., which found the prevalence of PH in 1250 PLWH to be 17.4% [23].

The study of HIV-associated pulmonary vascular complications ideally requires a strong understanding of pulmonary vascular cell biology and HIV pathogenesis, which is at the crossroads between infectious diseases and vascular medicine. This review is intended to provide key background information in both research areas, highlight the current gaps in understanding, and provide potentially hypothesis-generating discussions regarding the impact of HIV on pulmonary vasculature. Understanding these factors is crucial, as it necessitates a comprehensive grasp of both pulmonary vascular cell biology and HIV pathogenesis, bridging the disciplines of infectious diseases and vascular medicine.

## 2. Phenotypic and Functional Properties of Pulmonary Vascular Endothelial and Smooth Muscle Cells

The pulmonary vasculature, like other blood vessels, comprises distinct layers with specialized cell types. These layers include the tunica intima, tunica media, and tunica adventitia, each associated with specific cell types: endothelial cells, smooth muscle cells, and fibroblasts, respectively [24,25]. Within the pulmonary arterial system, endothelial cells and smooth muscle cells primarily regulate blood pressure [26].

Endothelial cells line the inner surface of blood vessels and act as a vital barrier between the bloodstream and lung tissue [27–29]. They facilitate gas exchange in the alveoli and mediate key processes including vascular tone, the recruitment of inflammatory cells to the vasculature, vasoconstriction, and relaxation by expressing various surface receptors and adhesion molecules [27]. Despite their crucial function, endothelial cells exhibit remarkable diversity within the pulmonary vasculature. Recent advancements in single-cell RNA sequencing have uncovered the heterogeneity of human endothelial cells in the lungs [30]. Different subtypes of endothelial cells, including arterial, capillary, lymphatic, and venous, have been identified based on distinct genetic markers [30]. For instance, the arterial endothelial cells are classified according to the expression of EFNB2, SOX17, BMX, SEMA3G, HEY1, LTBP4, FBLN5, GJA4, and GJA4 [30]. RGCC, SPARC, SGK1, CA4, and PRX are canonical markers used to identify capillary endothelial cells (Table 1). The venous endothelial cell subtype is classified according to the expression of NR2F2, VCAM1, ACKR1, and SELP [30] (Table 1). Marker genes associated with the lymphatic endothelial cell type include PROX1, LYVE1, FLT4, and PDPN [30] (Table 1). Other common cell surface markers associated with vascular endothelial cells specifically include ICAM-2, CD105, VCAM-1, CD141, VE-cadherin, MCAM, CD201, CD248, CD309, CXCL16, ICAM-1, Tie-2, and VG5Q [31] (Table 1).

PH can result from the vasoconstriction of pulmonary smooth muscle cells and the proliferation of pulmonary endothelial and smooth muscle cells. Some traditional therapies for the treatment of PH include prostacyclin, calcium channel blockers, and nitric oxide [32]. Prostacyclin is a vasodilator and one of the most effective drugs for the treatment of pulmonary hypertension [33]. Calcium channel blockers work by preventing calcium from entering cells and therefore preventing contraction but are not effective in all idiopathic pulmonary arterial hypertension (IPAH) patients [34]. Inhaled nitric oxide regulates vascular tone and causes vascular smooth muscle cells to relax [35]. For PLWH, drug interactions between their PH treatment and their antiretroviral drug regimen must also be considered [36]. Markers of endothelial dysfunction such as endothelin-1, which plays a role in smooth muscle proliferation, are elevated in PH patients and serve as therapeutic targets [37]. Endothelin receptor antagonists inhibit the binding of endothelin-1 and improve the progression of PH. Therapies that reverse or prevent the vasoconstrictive and proliferative mechanisms associated with PH are preferable, and several markers, molecules, and genetic factors have been explored as therapeutic targets (Table 1).

In addition to their heterogeneity, endothelial cells influence the function of underlying smooth muscle cells. Vascular smooth muscle cells, responsible for vessel stability through constriction and relaxation, contribute to maintaining vascular homeostasis and appropriate blood pressure levels [56–58]. These cells also exhibit diversity, with contractile and synthetic smooth muscle cells serving different functions within the pulmonary vasculature. While contractile smooth muscle cells display elongated, spindle-shaped morphology, synthetic smooth muscle cells resemble cobblestones [59]. The contractile smooth muscle cell type is associated with marker proteins such as the α-smooth muscle actin, smooth muscle-myosin heavy chain, SM22α, SM-calponin, H-caldesmon, smoothelin, telokin, and meta-vinculin [56] (Table 2). The synthetic smooth muscle cell type can be identified by a lack of markers that are associated with the contractile smooth muscle cell type. In addition, osteopontin, vimentin, matrix metalloprotease-9, and epiregulin are highly expressed in synthetic vascular smooth muscle cells, as reported by several studies [60–62]. Ideally, smooth muscle cells are distinguished by multiple factors that complement each other such as the analyzation of morphology and the presence of marker proteins [56]. The extensive proliferation of smooth muscle cells leads to pulmonary vascular remodeling. This involves vascular medial thickening and the contraction of smooth muscle cells, which makes the cells more susceptible to hypoxia and inflammation [63]. There are transcription factors and pathways associated with the smooth muscle cell proliferation that occur in PH such as the Janus kinase (JAK)/signal transducer and the activator of transcription (STAT) pathway [64]. Blocking this pathway with ruxolitinib therapy has been explored as a potential therapeutic option for PH patients and it has been shown to attenuate elevation in pulmonary arterial pressure and reduce vascular remodeling [65]. Hypoxia-inducible transcription factors have also been explored as therapeutic options for pulmonary vascular remodeling since they regulate oxygen homeostasis and hypoxic adaptation. The modification of the CD146/Hypoxia Inducible Factor 1 alpha (HIF-1α) pathway has been shown to reduce vascular remodeling [66]. Transcriptional factors involved in the mitochondria-dependent apoptosis, hypertrophy, and pyroptosis of pulmonary arterial smooth muscle cells have also been explored as therapeutic options for vascular remodeling. This includes transcription factors Nogo-B, NFATc3, and KIF23, respectively [67–69].

Healthy endothelial cells interact with smooth muscle cells to regulate vasoconstriction and maintain normal blood flow [70]. However, disruptions in this interaction can lead to pathological conditions such as pulmonary hypertension, as evidenced by studies such as that by Asosingh et al., which highlighted altered gene expression in pulmonary artery endothelial cells from individuals with pulmonary hypertension [71]. The communication and coordination between endothelial and smooth muscle cells are essential for preserving pulmonary vascular health and function.

### The Role of Pulmonary Vascular Tone in Health and Disease

The pulmonary vasculature operates within a complex network of cells, including epithelial cells, nerve cells, immune cells, endothelial cells, and mesenchymal cells, each contributing to its physiological processes [72]. Central to the regulation of pulmonary vascular function is the concept of vascular tone, which signifies the equilibrium between the relaxation and contraction of vascular smooth muscle cells within small arteries and arterioles [73]. This balance is crucial for ensuring adequate oxygen delivery to support cellular metabolism and physiological functions [74].

In humans, oxygen from the environment binds to circulating hemoglobin and is transported through the cardiovascular system to reach tissues. The ventilation-to-perfusion ratio, which refers to the matching of ventilated regions of the lung with areas receiving adequate blood supply (perfusion), ideally remains around 1 in healthy individuals [75–77]. However, in hypoxic environments where oxygen levels drop below 2–6%, the lung employs hypoxic pulmonary vasoconstriction as a mechanism to redistribute blood flow to well-oxygenated regions [78–80]. This physiological response enhances ventilation-to-perfusion matching and facilitates gas exchange within a range of 0.01 to 1.0 [81].

Hypoxic pulmonary vasoconstriction is mediated through various signaling mechanisms, including paracrine signaling, direct cell-to-cell contact, via extracellular vesicles that are secreted by nearly every cell type within the lung, or cargo nucleic acids, protein, lipids, or carbohydrates [82,83]. In addition, paracrine vascular signaling is aided by macromolecular networks provided by the extracellular matrix (including fibronectin, integrin, non-integrin receptors, and growth factors), which allow them to communicate with the extracellular environment [83,84].

Endothelial cells play a crucial role in orchestrating vascular tone through the production of mediators such as nitric oxide, prostacyclin, and endothelin-1 [85]. Pulmonary vasoconstriction is a biological mechanism that, in healthy individuals, redirects blood in a way that optimizes ventilation–perfusion matching [86]. However, the dysregulation of endothelial cell function can lead to abnormalities in vascular tone and contribute to the pathogenesis of pulmonary vascular diseases. Markers for endothelial cell dysfunction include the localized mediators nitric oxide, prostacyclin, and endothelin-1 as well as VCAM-1, ICAM-1, and MCP-1 (Table 1) [87,88]. However, the levels of these mediators change under hypoxic conditions. Hypoxia in vitro is typically achieved under 1% oxygen [89], while 5–10% oxygen levels *in vivo* are accepted as hypoxic [90,91].

Under hypoxic conditions, endothelial cells utilize nitric oxide, prostacyclin, and endothelin-1 to induce contraction in vascular smooth muscle cells, leading to vasoconstriction. This process involves a cascade of events, including membrane depolarization, increased cytosolic calcium concentration, and the activation of myosin light chain kinase [87,92,93]. Dysfunction in vascular smooth muscle cells, characterized by vessel wall thickening and alterations in intraluminal diameter, further exacerbates pulmonary vascular abnormalities [85,88].

**Figure 1.**
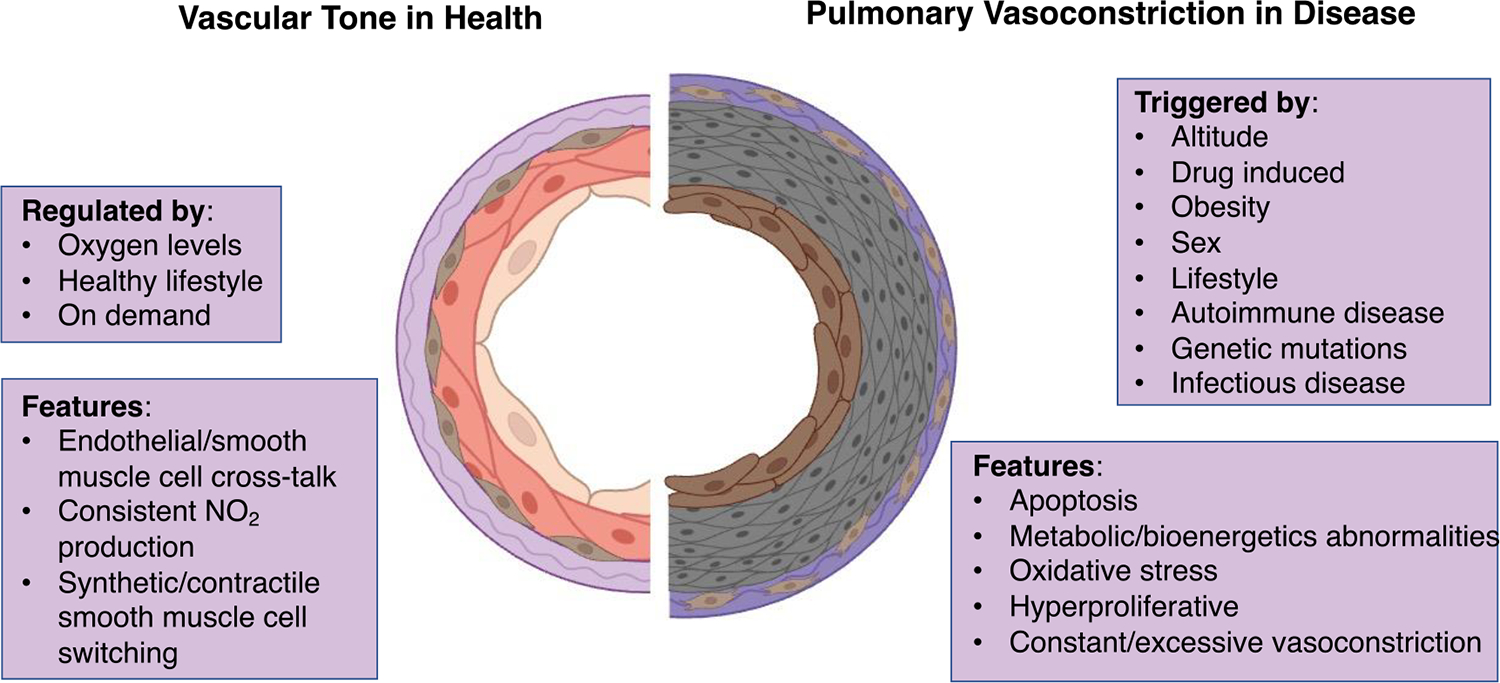
Schematic of healthy vascular tone compared to diseased pulmonary vasoconstriction. Several factors including lifestyle, oxygen levels, and infectious diseases can influence vascular tone. Features of healthy vascular tone include nitric oxide production, as well as cell cross-talk between endothelial, smooth muscle cells, and adventitial cells. Features of diseased pulmonary vasoconstriction include apoptosis, oxidative stress, and hyperproliferative cells.

Various stimuli, including the tissue partial pressure of oxygen in the pulmonary arteriole, trigger pulmonary vessel constriction (Figure 1) [87]. The mechanisms through which pulmonary artery smooth muscle cells sense oxygen levels involve mitochondrial reactive oxygen species production, cellular energy status, the activation of hypoxia-inducible transcription factors, and changes in cytoplasmic redox state [87].

The delicate balance between vascular tone and constriction is pivotal for maintaining pulmonary vascular health. The dysregulation of these processes leads to pathological conditions, with several infectious pathogens, including HIV, implicated in triggering pulmonary vessel constriction and contributing to disease progression.

## 3. Pulmonary Vascular Concerns in People Living with HIV

The advent of cART has transformed HIV into a chronic condition by suppressing viral replication, thereby prolonging the lifespan of PLWH [94]. However, this prolonged lifespan has been accompanied by an increased susceptibility to various comorbidities, including diabetes, dyslipidemia, chronic kidney disease, metabolic disorders, neuropathies, and cardiovascular diseases [95–97].

Pulmonary vasoconstriction emerges as a significant concern among PLWH due to the heightened risk of developing life-threatening pulmonary diseases including pulmonary hypertension (PH), acute respiratory distress syndrome (ARDS), pulmonary embolism, and chronic obstructive pulmonary disease (COPD) [36]. In general, chronic inflammation, endothelial dysfunction, and oxidative stress are some of the factors that contribute to the susceptibility to pulmonary vasoconstriction, all of which are observed in HIV [98]. Conditions such as COPD and non-infectious respiratory diseases like asthma and cardiopulmonary dysfunction can exacerbate the impact of pulmonary vasoconstriction in PLWH [98]. The resultant excessive vasoconstriction often leads to increased pulmonary vascular resistance, a hallmark of pulmonary hypertension, a condition significantly more prevalent in PLWH [99,100].

Despite the increased risk, screening for severe pulmonary hypertension through ultrasound or diagnosis via right heart catheterization is not part of the standard of care for PLWH. PLWH do have recommendations to receive echocardiographic screening for PH if they have any of the following risk factors: intravenous drug use, female sex, hepatitis C virus infection, come from a high-prevalence country, or are US African American [101]. In 2022, The European Society of Cardiology/European Respiratory Society defined these criteria as having a >20 mm Hg mean pulmonary artery pressure, a pulmonary wedge pressure of ≤15 mm Hg, and a pulmonary vascular resistance of ≥2 Wood Units [102], which are determined by means of invasive right heart catheterization [103]. While the diagnostic tools used to identify pulmonary hypertension are the same regardless of the cause, how HIV (or its proteins) increases the susceptibility to pulmonary vascular disease remains enigmatic.

## 4. Role of HIV Proteins in Pulmonary Vascular Diseases

HIV is a bloodborne pathogen, which guarantees consistent interactions with vascular cells across various tissues. The infection mechanism of HIV involves fusion with the cellular membrane of susceptible cells, allowing entry and subsequent replication within the host [104,105]. While HIV predominantly targets CD4+ T cells and macrophages [106], studies with HIV and its simian counterpart SIV have shown its ability to infect non-immune cells such as bronchial epithelial cells, smooth muscle cells, and pericytes *in vivo* [107–109].

The damage to the immune system caused by the destruction of lymphocytes during HIV infection impairs host immune function. During acute infection, there is a depletion of CD4+ T cells and large pools of infected CD4+ T cells in the blood and lymphoid tissues [110]. Humoral and cell-mediated host immune responses occur during peak viremia [111]. Activation-induced cell death, viral cytopathic effects, or cytotoxic T lymphocyte-mediated cell killing are the primary causes of death in many CD4+ T cells [111]. CD4+ T cell numbers continue to decrease, and viremia levels stabilize during chronic infection, resulting in chronic immune activation and inflammation [112]. Although this immune dysfunction leads to opportunistic infections, the development of HIV-PH appears to result from right ventricular dysfunction rather than immune deficiency [113]. However, PH further complicates infection with HIV and is associated with defective CD4+ T cells. Several inflammatory processes have been identified as pathogenic components of pulmonary vascular remodeling and have been linked to PH [114]. Natural killer (NK) cells, which are innate lymphocytes with the ability to identify and eliminate cells infected with viruses, have been reported to produce increased amounts of matrix metalloproteinases in patients with PAH [115]. This causes functional damage to NK cells and affects vascular remodeling [116]. Functionally damaged NK cells also result in the increased production of IL-23. CD8+ T cells have been identified as an important element in the inflammatory response seen in vascular remodeling. PAH patients who are deficient in CD8+ T and NK cells have been recognized as having an increased risk of death [117]. Increased numbers of CD68+ and CD14+ macrophages, CD3+ T cells, CD8+ T cells, and CD4+ helper T cells have been found in vessels of lungs from patients with PAH [118].

HIV belongs to the lentivirus family and its RNA genome encodes for 15 viral proteins within nine open reading frames [119]. The RNA genome is surrounded by matrix proteins between the lipid envelope and viral capsid and enclosed within the viral core [120]. The viral capsid contains enzymes necessary for viral replication, such as reverse transcriptase, integrase, and proteases, coded by the viral *pol* gene [121]. The viral reverse transcriptase converts the HIV RNA into DNA upon virus entry into the host [122,123]. Viral DNA is then incorporated into the host DNA using integrase, creating a permanent copy of the viral DNA in the cell [123,124]. It is important to emphasize that the integration of viral DNA into the host chromosomes ensures the lifetime persistence of HIV until a definitive cure is achieved.

The viral proteins play pivotal roles in viral replication and pathogenesis. For instance, HIV Gag is a polyprotein precursor that plays a crucial role in the assembly and release of new HIV virions. The Gag polyprotein is cleaved by the viral protease into individual structural proteins, including matrix (MA), capsid (CA), nucleocapsid (NC), and p6 proteins, which are essential for the formation of mature infectious viral particles. Gag is involved in directing the assembly of viral structural proteins and genomic RNA at the plasma membrane of the host cell, aiding in the formation and release of new virus particles. The Gag protein is also involved in the selection and packaging of viral RNA during virus assembly. Additionally, HIV Gag interacts with cellular factors and membrane components to facilitate the budding and release of new virions [125]. While structural HIV proteins are commended for ensuring the integrity of the virus, other HIV-encoded proteins like Tat, Nef, and gp120 may unarguably be considered the three most pathogenic proteins.

### 4.1. HIV Tat (Trans-Activator of Transcription)

**Figure 2.**
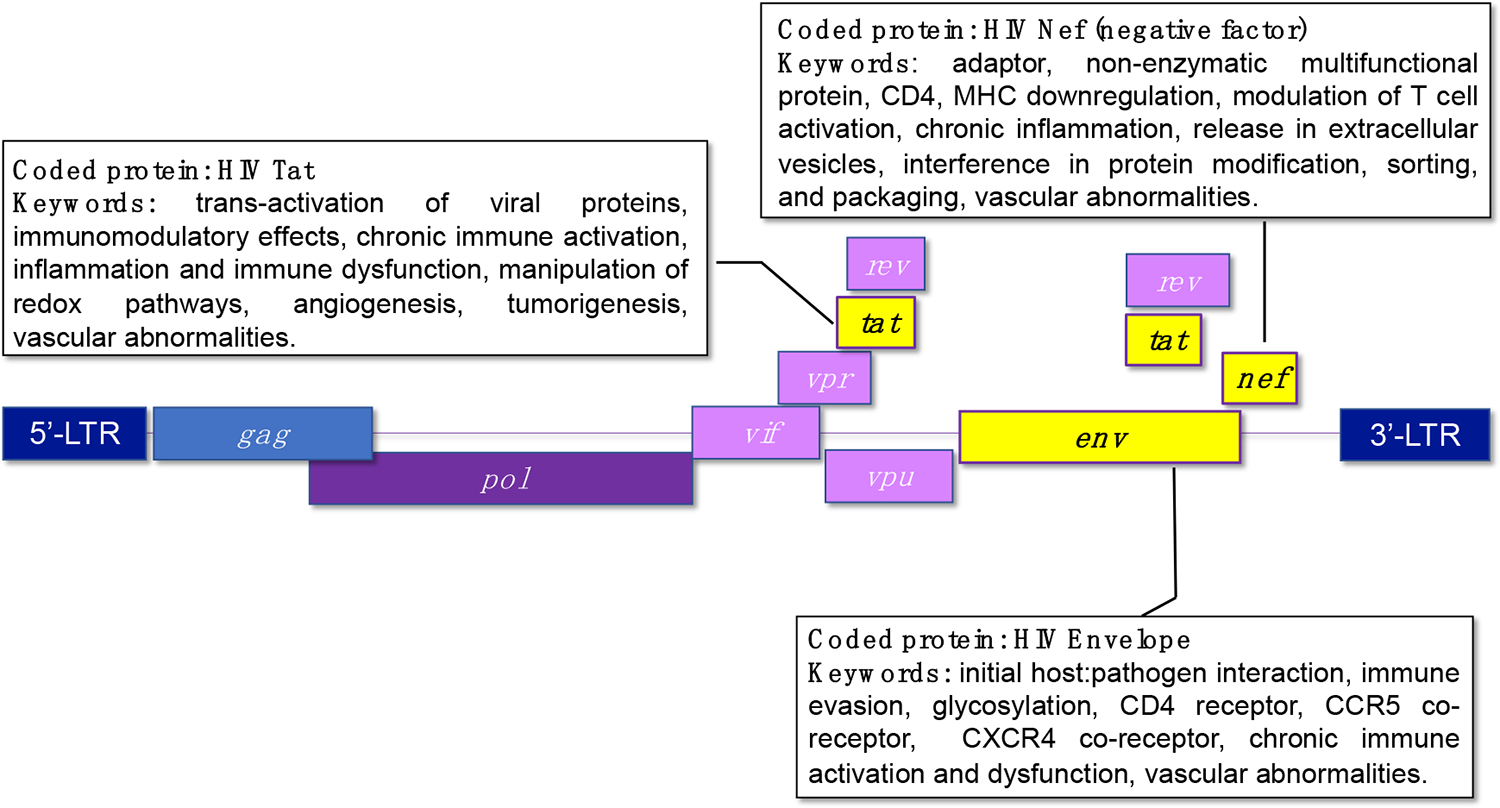
Overview of HIV-1 *tat, env*, and *nef* in the viral genome including their function and role in the vasculature. HIV-1-encoded proteins Nef, Tat, and gp120 play roles in the damage of the vascular endothelium that contributes to vascular diseases in people living with HIV.

Tat is a non-structural regulatory viral protein that plays a crucial role in the viral life cycle and pathogenesis through the trans-activation of viral genes (Figure 2). Tat regulates HIV gene expression by binding to the trans-activation response (TAR) element located at the 5’ end of viral RNA transcripts. By interacting with cellular transcription factors and the RNA polymerase II complex, Tat enhances the processivity and efficiency of viral transcription, leading to an increased production of viral RNA and proteins. Tat enhances viral transcription and promotes the replication of HIV within infected cells. In addition, Tat interacts with various immune cells and signaling pathways. Tat activates T cells and macrophages, promotes the production of pro-inflammatory cytokines and chemokines, and alters the function of dendritic cells and natural killer cells. These immunomodulatory effects can contribute to chronic immune activation, inflammation, and immune dysfunction in HIV-infected individuals. Conceivably, Tat is implicated in the pathogenesis of HIV-associated neurocognitive disorders (HAND) by promoting neurotoxicity and neuronal damage. Tat crosses the blood–brain barrier and directly affects the function of neurons and glial cells in the central nervous system. Tat-induced neurotoxicity is mediated by various mechanisms, including oxidative stress, mitochondrial dysfunction, and the dysregulation of calcium homeostasis. Tat is secreted from HIV-infected cells and can be detected in the sera of HIV-infected patients, which then causes inflammation and damage in non-infected (bystander) cells. For example, Lee et al. have demonstrated that Tat can produce mRNA and protein expression of IL-1β, a pro-inflammatory cytokine, in human peripheral vascular endothelial cells [126]. Furthermore, HIV Tat modulates cellular gene expression, manipulates redox-modulating pathways, stimulates the expression of proangiogenic factors, and promotes the proliferation and migration of endothelial cells and tumorigenesis. This contributes to the development of dilated cardiomyopathy, arrhythmias, atherosclerosis, and pulmonary hypertension among other vascular abnormalities and AIDS-related malignancies [127–129]. To our knowledge, HIV Tat was first associated with PH shortly after mutations in the bone morphogenetic protein receptor-2 (BMPR-2) were associated with heritable PH, in studies that demonstrated that Tat repressed the BMPR-2 promoter in transfected macrophage cell lines [130].

### 4.2. HIV Nef

The HIV protein Nef (negative factor), coded by the HIV *nef* gene, is a regulatory, non-enzymatic accessory multifunctional adaptor protein that is essential for viral replication, immune evasion, and the modulation of host cell functions (Figure 2). Nef has well-established roles in HIV pathogenesis, including the downregulation of CD4 and major histocompatibility complex class I (MHC) molecules, the enhancement of viral infectivity, the modulation of T cell activation, and the evasion of host immune responses.

Why would HIV downregulate the receptors used for infection? Nef promotes the internalization and degradation of CD4 molecules from the surface of infected cells. By reducing CD4 expression, Nef enhances the release of viral particles from infected cells, which enhances the infectivity and dissemination of HIV within the host. CD4 downregulation also prevents superinfection by blocking the CD4-mediated entry of other HIV particles. Additionally, Nef downregulates MHC-I molecules, impairing the presentation of viral antigens to cytotoxic T lymphocytes and evading immune detection. In addition, Nef alters various signaling pathways in infected T cells, leading to the dysregulation of immune responses. Nef can activate signaling molecules such as Src-family kinases and phosphatidylinositol 3-kinase (PI3K), leading to changes in cell morphology, motility, and cytokine secretion. These alterations contribute to immune activation, T cell dysfunction and depletion (mostly by apoptosis), and the progression of HIV infection and HIV-associated complications, including AIDS-related illnesses and HIV-associated neurocognitive disorders.

Nef induces endothelial apoptosis and dysfunction, exacerbating pulmonary vascular diseases [131,132]. Mechanistically, Nef activates GTPase Rac1, leading to the generation of reactive oxygen species, which contribute to endothelial dysfunction [131]. Notably, Nef can persist in the lung, potentially inducing vascular disease in patients receiving cART.

### 4.3. HIV gp120

**Figure 3.**
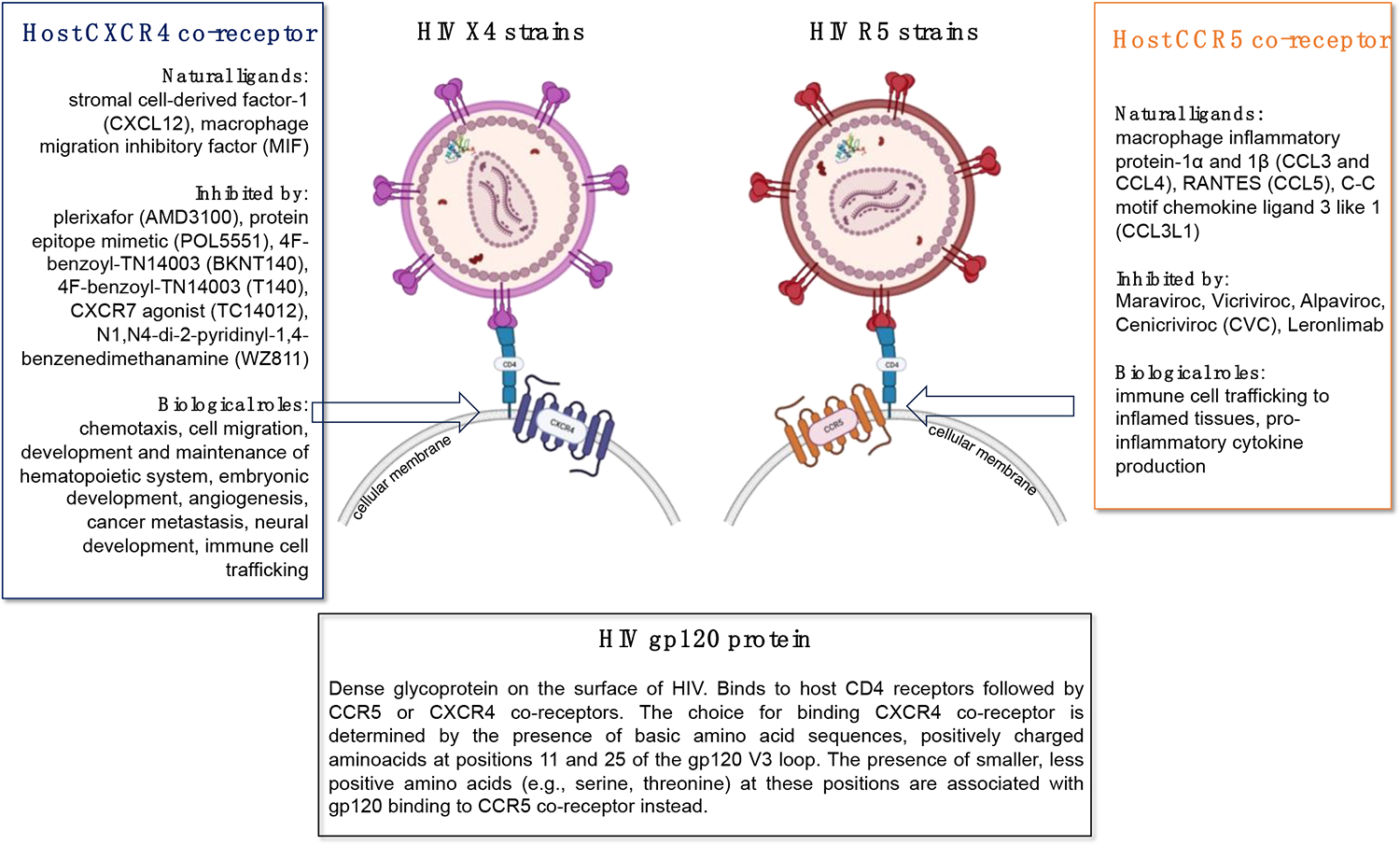
Diagram indicating HIV X4, which uses C-X-C chemokine receptor type 4 (CXCR4) and HIV R5, which uses C-C chemokine receptor type 5 (CCR5). Interactions with CXCR4 or CCR5 are determined by HIV-1 glycoprotein 120 (gp120).

The HIV glycoprotein-120 is a glycan-rich viral protein with a molecular weight of 120 kilodaltons (hence, its name), that plays several crucial roles in the HIV lifecycle and infection process including the initial attachment of the virus to host cells, the mediation of viral entry, immune evasion, and the induction of host cell signaling (Figure 2). As an obligated intracellular parasite, HIV must enter a susceptible cell as the first step in the infection process. HIV gp120 is responsible for binding to specific host cell surface CD4 receptors present mostly on helper T cells, macrophages, dendritic cells, and monocytes. This initial attachment step is essential for the subsequent fusion of the viral and host cell membranes. After binding to the host CD4 receptor, HIV gp120 undergoes a conformational change that allows it to interact with a co-receptor on the host cell surface, typically either the C-C chemokine receptor type 5 (CCR5) or the C-X-C chemokine receptor 4 (CXCR4) (Figure 3). This interaction triggers further conformational changes in the viral envelope glycoproteins, leading to the fusion of the viral and host cell membranes and the entry of the viral core into the host cell cytoplasm. HIV gp120 is highly variable and heavily glycosylated, which works favorably for the virus to evade host immune surveillance. The extensive glycosylation of gp120 can shield it from recognition by neutralizing antibodies, allowing the virus to escape immune detection and continue infecting host cells. It is important to emphasize that regardless of whether HIV is able to successfully infect a cell or not, the binding of gp120 to CD4 and co-receptors can trigger signaling pathways within the host cell, leading to cellular activation. Consequently, HIV gp120 binding to host cell receptors can induce the production of pro-inflammatory cytokines and chemokines, contributing to chronic immune activation and inflammation.

HIV gp120 is implicated in lung endothelial cell apoptosis and endothelial dysfunction, potentially contributing to chronic lung diseases such as pulmonary emphysema [133,134]. Gp120 upregulates the expression of pro-inflammatory cytokines and peptides associated with vascular remodeling, further exacerbating vascular damage in PLWH. Green et al. have demonstrated that gp120 upregulates the cell surface expression of pro-inflammatory cytokines EMAP II and CXCR3, its receptor, which have been shown to contribute to lung endothelial apoptosis [133]. Kanmogne et al. demonstrated increased endothelin-1 secretion in primary human lung microvascular endothelial cells. Since endothelin-1 is a peptide associated with vascular remodeling, their findings suggest that gp120 may contribute to HIV-associated severe vascular damage [134]. Moreover, studies have shown that HIV gp120 variants may play a role in the pathogenesis of pulmonary arterial hypertension, with specific variants altering gene expression patterns in pulmonary artery endothelial cells [135–137]. These variants exhibit differential effects on host cell proteins, potentially contributing to the diverse complications observed in HIV patients [137].

## 5. Genetic Diversity of HIV: Implications in Virus Adaptation and Onset and Progression of Pulmonary Vascular Disease

One of the defining features of HIV is its high genetic diversity owing to several factors including its high mutation rate, recombination between viral strains, immune selective pressures, and even geographical factors. HIV has a high mutation rate (with 10^−3^ to 10^−5^ mutations per base pair [138–142]), due to the error-prone nature of its reverse transcriptase enzyme, which lacks proofreading activity. As a result, during the process of reverse transcription, errors frequently occur, leading to the generation of numerous genetic variants, or quasispecies, within the viral population. In addition, HIV can undergo genetic recombination when two different viral strains infect the same host cell. During reverse transcription, the viral RNA genomes from different strains can mix and recombine, resulting in the generation of recombinant viruses with unique genetic compositions. Furthermore, the human immune system and antiretroviral drugs exert selective pressure on the virus, favoring the survival and replication of variants that can evade immune detection or resist drug inhibition. This selective pressure drives the emergence of drug-resistant strains and immune escape mutants, contributing to the overall genetic diversity of HIV. Moreover, HIV exhibits geographic and epidemiological patterns of diversity, with distinct viral subtypes and recombinant forms prevalent in different regions of the world.

The genetic diversity of HIV guides its classification into several groups, subtypes (or clades), and circulating recombinant forms (CRFs). Essentially, HIV is divided into two main groups: HIV-1 and HIV-2. HIV-1 is responsible for the vast majority of HIV infections worldwide, which is further divided into multiple subtypes and CRFs. HIV-2 is less prevalent and primarily found in West Africa, although it is also present in other regions. HIV-1 is further classified into different subtypes based on genetic differences in the viral genome. The main subtypes of HIV-1 include subtypes A, B, C, D, F, G, H, J, and K. Each subtype is characterized by distinct genetic sequences and geographical distribution patterns. For example, subtype B is predominant in Western countries, while subtype C is prevalent in Southern Africa and India. In addition to subtypes, HIV-1 also forms CRFs through genetic recombination between different subtypes. CRFs contain genetic sequences derived from two or more HIV-1 subtypes and represent unique recombinant viruses with distinct genetic profiles. Examples of CRFs include CRF01_AE, which is prevalent in Southeast Asia, and CRF02_AG, which is common in West and Central Africa. Apart from CRFs, HIV-1 can also give rise to unique recombinant forms (URFs) when recombination events occur between different subtypes in individual patients. URFs represent rare recombinant viruses that do not fit into any established CRF category. Additionally, there is a rare group of HIV-1 viruses known as group O, which is distinct from the main group M (major) and group N (non-M, non-O) viruses. Group O viruses are mainly found in West and Central Africa and represent a minor proportion of HIV infections globally. HIV variants exhibit distinct geographic distribution patterns, with certain subtypes or recombinant forms being more prevalent in specific regions [143–145]. Differences in regional epidemiology and healthcare infrastructure may impact access to diagnosis, treatment, and care, leading to disparities in disease outcomes.

The intrinsic HIV genetic diversity has several important implications for pathogenic outcomes of infection and the overall battle to combat HIV. For instance, the efficiency of HIV transmission can vary between different viral variants. Some subtypes or recombinant forms may be more transmissible than others, leading to differences in the spread of infection within populations. Certain HIV subtypes or recombinant forms may be associated with different rates of disease progression. For example, some studies have reported that infection with subtype D or CRF02_AG may be associated with more rapid disease progression compared to other subtypes. The presence of diverse viral variants can complicate antiretroviral therapy by allowing for the emergence of drug-resistant strains. Monitoring viral diversity and drug resistance mutations is essential for optimizing treatment regimens and preventing treatment failure. HIV variants may differ in their ability to evade host immune responses or induce immune activation. Some variants may exhibit enhanced immune evasion mechanisms or modulate host immune responses in ways that affect disease progression and the immune control of the virus. The genetic diversity of HIV also poses challenges for vaccine development, as the virus can rapidly evolve to evade immune responses. Designing vaccines that provide broad and durable protection against diverse viral strains remains a major scientific challenge. Molecular epidemiological techniques, such as phylogenetic analyses, remain valuable for tracking the transmission and evolution of HIV within and between populations. Therefore, studying the genetic diversity of HIV still provides valuable insights into viral transmission dynamics, population movements, and the spread of drug-resistant strains.

The important roles of HIV genetic variations and pulmonary vascular diseases were uncovered by the characterization of the HIV *nef* gene in patients with diagnosed HIV-associated PH in a collaborative study leveraging samples from the French National Registry of PH and the HIV Cardiology clinic at the University of California in San Francisco (UCSF). The initial sequencing of the *nef* genes in the French cohort uncovered 10 mutations in several Nef functional domains, particularly in regions important for MHC-1/AP-1 binding sites and PKC phosphorylation [146]. Intriguingly, SHIVnef-infected monkeys and HIV-PH patients exhibited potentially impactful mutations in the N-terminus of the Nef polypeptide. First, the HIV-PH phenotype was associated with the substitution of an alanine for tyrosine at position 40, which may be important for the phosphorylation of SH3-containing proteins and PAK activation [146–148]. Second, an alanine-to-proline mutation at position 53 of Nef may bend the polypeptide to expose other additional motifs in the carboxy-terminal, in which additional mutations were identified in HIV-PH at the acidic cluster, CD4 downregulation domain, PKC phosphorylation site, and at the proline-rich PxxP motif [146].

## 6. Research Tools for Investigating HIV-Associated Pulmonary Vascular Comorbidities *In Vivo*

**Figure 4.**
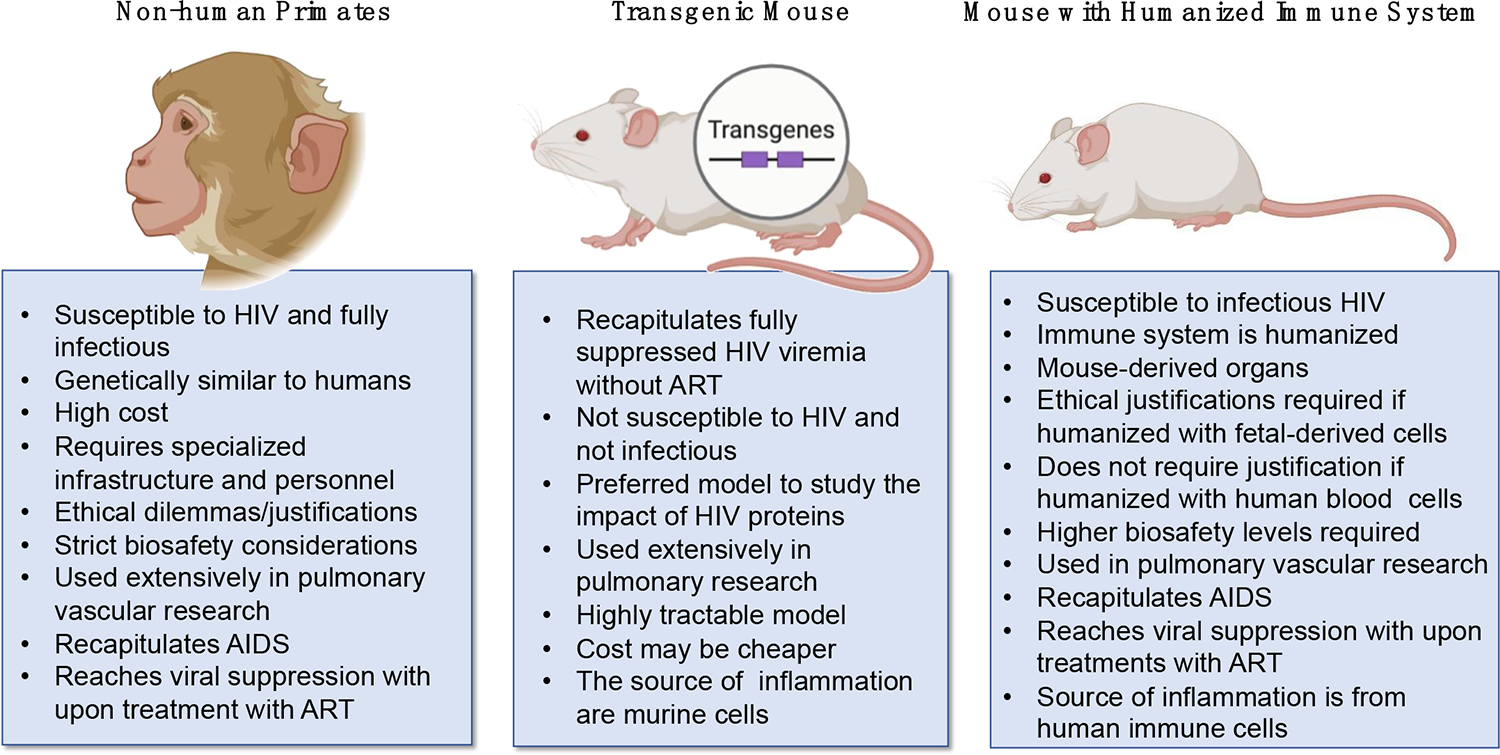
Comparison between animal models to study HIV-associated pulmonary vascular comorbidities.

Studying HIV-associated pulmonary vasoconstriction requires suitable animal models that recapitulate key aspects of HIV infection and its effects on the pulmonary vasculature. To study severe pulmonary vascular diseases such as PH that involve elevated pulmonary arterial pressure and resistance in the vasculature, it is difficult for one animal model to exhibit all remodeling that occurs [149]. An ideal animal model should be able to display an increase in mean pulmonary arterial pressure or right ventricular systolic pressure resulting from vascular remodeling and pulmonary vascular resistance [150]. To date, animal models such as mice, rats, cows, sheep, and monkeys have been used to study the pathogenesis of severe vascular diseases such as PH [151]; however, most of these models are not naturally susceptible to HIV infection. Currently, the two infectious models that have been developed to study molecular and physiological aspects of HIV-induced pulmonary vascular diseases are non-human primates (NHPs) and mice with humanized immune systems (hu-mice) (Figure 4).

### 6.1. Non-Human Primate Models of HIV Pathogenesis and Severe Pulmonary Vascular Remodeling

NHPs such as chimpanzees and macaques, particularly rhesus macaques (*Macaca mulatta*) and pig-tailed macaques (*Macaca nemestrina*), have advanced the understanding of HIV and simian immunodeficiency virus (SIV) pathogenesis tremendously, for over 3 decades [152]. As NHP hosts, several species of monkeys can become infected with Simian Immunodeficiency Virus (SIV), but not all of them develop clinical illness. Some monkeys serve as natural hosts for SIV and typically do not develop AIDS-like symptoms, while others may experience disease progression similar to HIV-infected humans. For example, African Green Monkeys (*Chlorocebus* spp.) and Sooty Mangabeys (*Cercocebus atys*) are natural hosts for SIV and are commonly infected with various strains of the virus in the wild. They typically do not develop AIDS-like symptoms and maintain high viral loads without progression to disease [153]. Non-natural hosts of SIV remain enlightening in the field of HIV pathogenesis, as they provide tools to understand the role of T cells in SIV/HIV disease. For instance, recent studies have established that T-cell depletion in the periphery does not affect gut integrity or drive disease progression but highlights systemic inflammation [154,155]. Conversely, non-natural hosts of SIV such as rhesus macaques, pig-tailed macaques, cynomolgus macaques (*Macaca fascicularis*), and others, are commonly used as experimental models for HIV/AIDS research. While macaques can become infected with SIV and develop AIDS-like symptoms under experimental conditions, they are not natural hosts for the virus.

Not only are the hosts important for HIV research, but also the pathogens used in experimental settings. Regarding the viral pathogen, both HIV and SIV are lentiviruses, members of the Retroviridae family, sharing similar genomic information and morphology [156], yet with differences. For example, SIV Tat and HIV Tat exhibit sequence diversity due to evolutionary divergence between the two viruses and adaptation to different host species [157]. SIV Tat and HIV Tat have evolved to function within their respective host species, reflecting adaptations to different cellular environments and immune responses. HIV-1 and some strains of HIV-2 encode a viral protein U (Vpu) that contributes to viral replication and immune evasion by antagonizing host restriction factors, such as tetherin, which inhibits viral release from infected cells. SIV, on the contrary, generally lacks a Vpu gene; however, certain SIV strains, such as SIVcpz (found in chimpanzees) and SIVgor (found in gorillas), encode a Vpu-like protein known as Vpu/gp2. In addition, Nef proteins from HIV and SIV share structural and functional similarities but there are differences in their specific activities and interactions with host cell proteins, reflecting evolutionary divergence between the two viruses. Given the intrinsic differences between SIV and HIV, the use of chimeric Simian–Human Immunodeficiency Viruses (SHIV) has enabled researchers to study the effects of specific HIV proteins in NHPs.

NHPs have successfully been used as models for studying not only HIV infection but its associated pulmonary complications. NHPs were instrumental in establishing the first associations of HIV protein Nef with severe pulmonary vascular remodeling. Rhesus macaques infected with chimeric SHIVnef exhibited significant pulmonary vascular remodeling and evidence of plexiform lesions that were indistinguishable from those in human PH [158,159]. HIV Nef co-localized to pulmonary artery endothelial cells in the macaques, which was confirmed in human pulmonary arteries. A further characterization of SHIV-nef-associated pulmonary vascular disease in separate rhesus macaques demonstrated the widespread accumulation of smooth muscle cells and stem cells, with prominent cell proliferation markers [160]. At the sub-cellular level, significant Golgi dysfunction in pulmonary arteries was demonstrated in SHIVnef- but not SIV-infected macaques [161,162]. These studies used the GOLGB1 (giantin), important in the preservation of structure and function of the Golgi apparatus, and responsible for protein modification, sorting, and packaging for intracellular or extracellular transport. The results showed that pulmonary arteries in SIV-infected macaques exhibited condensed fluorescent staining for giantin, indicative of intact Golgi apparatus. However, SHIV-nef-infected macaques with plexiform lesions demonstrated dispersed staining for giantin co-localizing with Nef-bearing endosomes, which was interpreted as Nef-associated severe Golgi dysfunction.

The associations of HIV proteins with severe pulmonary vascular remodeling using NHPs also involved HIV gp120. In this case, both rhesus macaques infected with SIV Δ670 and cynomolgus macaques infected with SHIVenv (89.6P) exhibited pulmonary arterial hyperplasia of medium and large vessels (62% in SHIVenv, 36% in SIV) and plexiform lesions [163]. Furthermore, longitudinal studies in rhesus macaques infected with SIV Δ670 uncovered three clinical outcomes in the SIV model, with half of the monkeys presenting PH post-infection, 28% with progressive PH, and 23% with transient PH [164].

### 6.2. Mice with Humanized Immune Systems (Hu-Mice) to Model HIV Diseases

Hu-mice represent the second model of infectious HIV *in vivo*. While mice have been used to study pulmonary complications for decades, it is critical to emphasize that rodents are not naturally susceptible to HIV infection because their cells lack the HIV receptors essential for infection. Therefore, the animals must be provided with susceptible cells for HIV infection after the complete suppression of the murine immune system. Hu-mice are immunodeficient and contain transplanted human hematopoietic stem cells (HSCs), peripheral blood cells, or lymphoid tissue [165]. Successful humanized mouse models lack murine T, B, and NK cells as a result of having the IL-2 receptor common gamma chain (IL-2rγ) deleted or inactivated in lymphopenic mice [151,166]. Lymphopenic-IL-2rγ deficient mice (non-obese diabetic/SCID-IL-2rγ^−/−^ or NSG mice) can be engrafted with human fetal liver and thymus and injected intravenously with HSCs [151] to generate the bone marrow–liver–thymus immune humanized mouse (hu-BLT) model, which has been established as an innovative *in vivo* system used to study HIV pathogenesis [167]. Hu-mice respond well to antiretroviral drugs tenofovir, emtricitabine, and raltegravir, which effectively inhibit HIV replication *in vivo* [168]. This model also successfully mimics levels of infected resting CD4+ T cells that are seen in patients receiving treatment with suppressive cART [168]. The NSG mouse model has been used in the development of new anti-HIV antibodies, which are of interest due to their potential use in antibody-based immunoprophylaxis and treatment [169]. More specifically, anti-HIV-1 gp120 monoclonal antibodies are of interest and the NSG model can imitate the human immune response to target HIV-1 gp120 [169].

Hu-mice have been used to model active HIV infection and associated pulmonary complications. Several mouse and rat models have prevailed as models of severe pulmonary vascular diseases like PH, including the hypoxia, monocrotaline, and Sugen (vascular endothelial growth factor receptor antagonist SU516) models or combinations [170–175]; however, none of these models are hosts for HIV in their natural form. Studies using NSG mice humanized with CD34+ hematopoietic stem cells or BLT infected intravenously with HIV NL43 and co-exposed with either hypoxia (10% measured oxygen in a normobaric chamber) or Sugen (injected subcutaneously every biweekly) demonstrated that humanized mice recapitulated PH-like and histopathologic and hemodynamic changes, with increased production of pro-inflammatory cytokines IL-8, IFN-g, TNF-a, IL-2, IL-13, IL-6, and IL-1b, with no significant changes in IL-12, IL-10 or IL-4 [151].

The use of infectious HIV animal models recapitulates the *in vivo* scenarios of HIV pathogenesis in the context of plasma viremia such as in acute and untreated infections, as well as antiretroviral drug failure. Of note, chronic HIV infection is achievable in NHP and hu-mice with antiretroviral drug treatments. Nevertheless, investigations utilizing infectious HIV animal models increase safety considerations for laboratory workers and may bring strains to research laboratories without optimal infrastructure for elevated biosafety levels. Hence, non-infectious transgenic models have offered successful alternatives to investigate HIV pathogenesis over decades.

### 6.3. Transgenic HIV Models

Non-infectious mouse and rat models have enlightened the field of HIV pathogenesis by mimicking the production of HIV proteins in the absence of active infection. Most HIV transgenic (Tg) models express all HIV proteins except Gag and Pol. For this reason, the virus is deemed non-infectious which makes it easier to handle in laboratory settings. HIV Tg male Fischer 344 rats [176,177] exhibit significantly increased right ventricular wall thickness, a thickening of the pulmonary arteries, a remodeling of the small pulmonary arteries, and elevated right ventricular systolic pressures, which was more predominantly in 9-month-old compared to 3-month-old rats [178]. In addition, these rats exhibited genetic expression profiles consistent with other models of PH, including a significantly increased transcription of endothelin-1 (ET-1), endothelial nitric oxide synthase (eNOS), bone morphogenetic protein receptor type 2 (BMPR-2), platelet-derived growth factor subunit B (PDGF-BB), platelet-derived growth factor receptor beta (PDGFR-β), hypoxia-inducible factor 1-alpha (HIF-1α), and vascular endothelial growth factor (VEGF). The HIV Tg models have also enlightened the field, uncovering exacerbations of pulmonary epithelial and vascular dysfunction in the context of drug abuse and co-infections. For example, chronic alcohol ingestion significantly decreased the liquid clearance of an intratracheal saline challenge in HIV Tg rats *in vivo* [179]. Alveolar epithelial cells derived from these alcohol-fed rats exhibited decreased paracellular permeability, correlating with the altered expression of the tight junction proteins occludin and ZO-1. The model also served to demonstrate that alcohol and HIV, alone or in combination, decreased the expression of antioxidant Nrf2 in the lungs [179]. Altogether, these results were interpreted as exacerbating the alveolar epithelial barrier dysfunction caused by chronic HIV-1-related protein expression. HIV Tg rats exposed to cocaine exhibited significantly increased mean pulmonary artery pressures and right ventricular systolic pressures, with the downregulation of BMP signaling and prominent pulmonary artery smooth muscle cell hypertrophy and apoptosis resistance [180]. Further studies using pulmonary arterial smooth muscle cells isolated from HIV transgenic rats and analyzed in vitro showed that cocaine and HIV Tat upregulated the production of microRNAs such as miR-216a that regulated BMP signaling and contributed to smooth muscle hyperplasia [181]. Regarding co-infections, HIV Tg26 mice served to demonstrate that HIV proteins decrease potassium channel currents in myocytes, with prominent decreases in depolarization pulses but no hemodynamic changes [182]. The two-hit approach applied to HIV Tg26 mice in the context of co-infection with *Schistosoma mansoni* was able to demonstrate significant pulmonary vascular remodeling and hemodynamic changes. In this model, HIV alone did not promote hemodynamic changes, and reduced the Fulton index, but was sufficient to increase the medial wall thickness of small pulmonary arteries. Animals co-exposed to HIV and *S. mansoni* eggs showed exacerbated pulmonary perivascular fibrosis and obliterative pulmonary vascular lesions [183], which were not observed in the HIV-hypoxia or HIV-Sugen infectious models [151].

## 7. Concluding Remarks and Perspectives

HIV not only directly impacts the immune system but also induces a chronic inflammatory state in people living with HIV. This chronic inflammation is the basis for several comorbidities that arise from the dysfunction of the vasculature at the end-organ level. In the lungs, chronic vascular dysfunction potentially results in serious conditions that are hard to diagnose in PLWH, such as PH or pulmonary thromboembolism (PTE), particularly because their screening is not part of the standard of care in HIV.

The role of HIV infection in pulmonary vascular dysfunction is fully recognized. The problem of exacerbated pulmonary diseases in HIV population is not new. However, the specific mechanisms underlying HIV-induced pulmonary vasoconstriction are not fully elucidated. While further research is needed to investigate the molecular pathways, cellular interactions, and signaling cascades involved in this process, decades of research keep piling and hundreds of scientists worldwide are working to increase awareness of this situation. Essentially, filling in the gaps in the understanding of HIV-associated pulmonary vascular diseases remains a work in progress.

Challenges in the field present themselves from two sides of the same coin: those imposed by the virus itself and those stemming from the host. On one side, the relentless mutant nature of HIV makes it a moving target that is hard to investigate universally, especially in the context of diverse geographical locations and exposure to highly heterogeneous antiretroviral regimens. Added complexities are introduced by aging, exposures to environmental triggers, and further damage caused by recreational or illegal drugs of abuse, as well as coinfections with bacterial, fungal, parasitic, or other viral pathogens. Limited studies have examined potential differences in the pathogenic outcomes of different HIV variants, including subtypes and recombinant forms, on pulmonary vascular function. Using the virus as a molecular biomarker, and understanding how specific viral genetic characteristics contribute to variations in pulmonary complications could inform targeted interventions.

On the other side of the coin, the interplay between HIV and host factors, such as genetic predisposition, immune status, and comorbidities, in the development of pulmonary vasoconstriction requires further investigation. Identifying host factors that modulate susceptibility to HIV-associated pulmonary complications should refine current risk stratification and personalized treatment approaches. To this end, more strategic leverage of retrospective and prospective samples collected from HIV-infected patients at risk of developing or already diagnosed with severe pulmonary vascular disease is crucial for the assessment and categorization of collective biomarkers that can be used for prevention purposes.

The study of HIV-associated pulmonary vascular complications ideally requires a deep understanding of pulmonary vascular cell biology and HIV pathogenesis, which is at the crossroads between infectious diseases and vascular medicine. Ideally, experts in HIV medicine should work closely with pulmonary specialists for more comprehensive approaches to managing HIV-associated pulmonary vascular comorbidities. In addition, physicians should ideally work in conjunction with scientists to further gain insights into mechanisms underlying pulmonary vascular cell biology.

The field may be advanced by further developing *in vivo* and in vitro models that are well described for both pulmonary hypertension and HIV for mechanistic studies. It would be ideal to utilize smaller animal models to favor reproducibility by investigators with no access to NHP facilities and alleviate ethical concerns associated with the use of NHPs. In addition, while important research efforts focus on the identification of novel therapeutic targets, revisiting most of the studies discussed in this review article calls for the repurposing of currently existing drugs that may be considered to further supplement current HIV antiretroviral therapy without drug interference or added drug toxicities.

In essence, the study of HIV-associated pulmonary vascular complications requires a multidisciplinary approach, drawing upon insights from both infectious diseases and vascular medicine. Bridging the gap between preclinical research findings and clinical practice is essential for improving patient care. Addressing these knowledge gaps requires interdisciplinary collaborations, innovative research approaches, and dedicated efforts to prioritize HIV-related pulmonary complications on the global research agenda.

## Figures and Tables

**Table 1.** Pulmonary vascular endothelial cell surface markers and cell adhesion molecules. Endothelial cells regulate essential vascular processes including vascular tone, the recruitment of inflammatory cells to the vasculature, vasoconstriction, and relaxation by expressing various surface receptors and adhesion molecules. Various subtypes of endothelial cells including arterial, capillary, lymphatic, and venous are now distinguishable and classified according to several molecular markers listed below [30,31].

Marker	Name	Description	Associated Endothelial Cell Type
ICAM-1	Intercellular adhesion molecule 1	The protein encoded by ICAM-1 serves as a surface glycoprotein expressed on endothelial cells and immune system cells such as natural killer cells and dendritic cells. ICAM-1 is a therapeutic target for bosentan, which reduces inflammation in PH patients by decreasing ICAM-1 and interleukin-6 levels in blood [38–40].	Canonical EC
ICAM-2	Intercellular adhesion molecule 2	ICAM-2 mediates adhesive interactions important for antigen-specific immune response, NK-cell mediated clearance, lymphocyte recirculation, and other cellular interactions important for immune response and surveillance [40].	Canonical EC
PECAM-1/CD31	Platelet and endothelial cell adhesion molecule 1	Protein encoded by PECAM-1 makes up endothelial cell intercellular junctions and contributes to the inflammatory cell accumulation that occurs during intima-media thickening and vascular remodeling. High levels of PECAM-1 are a potential indicator of response to therapeutic agent treprostinil [40,41].	Canonical EC
CD105	Endoglin	The CD105 gene enables protein homodimerization and transforming growth factor beta binding activity [40].	Canonical EC
VCAM-1/CD106	Vascular adhesion molecule 1	Ligands bind to this molecule on endothelial cell surfaces during inflammatory responses, mediating the adhesion of immune cells to vascular endothelium. The blocking of VCAM1 could serve as a potential therapeutic target because it has been shown to improve angiotensin II-induced hypertension and vascular dysfunction in mice [40,42].	Canonical EC
CD141	Thrombomodulin	This gene encodes an endothelial-specific type I membrane receptor that binds thrombin. CD141 is essential in the regulation of blood coagulation and inflammation [40,43].	Canonical EC
MCAM	Melanoma cell adhesion molecule	MCAM is involved in vascular wound healing and acts upstream of or within angiogenesis [40].	Canonical EC
CD248	Endosialin	CD248 is predicted to enable extracellular matrix binding activity and extracellular matrix protein binding activity. CD248 contributes to the remodeling of lung blood vessels that could result in PH [40,44].	Canonical EC
CD309	Kinase insert domain receptor	CD309 functions as the main mediator of VEGF-induced endothelial proliferation, survival, migration, tubular morphogenesis, and sprouting [40].	Canonical EC
CXCL16	C-X-C motif chemokine ligand 16	CXCL16 recruits and adheres to CXCR6. It is expressed on stimulated endothelial cells, smooth muscle cells, platelets, drndritic cells, and macrophages. It is involved in several processes, including cell growth, response to interferon-gamma, and response to tumor necrosis factor. In blood vessels, CXCL6^+^ endothelial cells recruit CXCR6^+^ T cells, and NK cells to vessel walls [40,45,46]	Canonical EC
Tie-2	TEK receptor tyrosine kinase	The ligand angiopoietin-1 (Ang-1) binds to this receptor and mediates embryonic vascular development. Rodents with constitutively expressed Ang-1 develop severe pulmonary hypertension. The Ang-1/Tie-2/serotonin pathway has shown therapeutic potential to treat PH because Ang-1 stimulates pulmonary arteriolar endothelial cells through the Tie-2 receptor. This results in the production of the smooth muscle mitogen serotonin, which is present in high levels in pulmonary hypertensive lung tissue from rodents [40,47].	Canonical EC
VG5Q	Angiogenic factor with G-patch and FHA domains 1	VG5Q encodes angiogenic factor AGGF1 that promotes the proliferation of endothelial cells [40].	Canonical EC
EFNB2	Ephrin B2	EFNB2 is a potent regulator of endothelial cell activity, and controls cell migration and angiogenesis. Pulmonary vascular remodeling has been found to be dependent on EFNB2-induced Eph receptor forward signaling in smooth muscle cells, making postnatal modifications of EFNB2-mediated intercellular communication a potential therapeutic option for pulmonary vascular remodeling [40,48].	Arterial EC
SOX17	SRY-box transcription factor 17	SOX17 participates in the regulation of embryonic development. The downregulation of levels of this gene in pulmonary arterioles leads to PH development when exposed to hypoxic conditions and there is therapeutic potential in the resulting upregulation of HGF/c-Met signaling [40,49].	Arterial EC
BMX	BMX non-receptor tyrosine kinase	BMX encodes non-receptor tyrosine kinase involved in signal transduction in the arterial endothelium [40].	Arterial EC
SEMA3G	Semaphorin 3G	SEMA3G is involved in endothelial cell migration [40].	Arterial EC
HEY1	Hes related family bHLH transcription factor with YRPW motif	HEY1 encodes nuclear protein that is a key downstream modulator of Notch signaling essential in cardiovascular development. Blocking the Notch-3-HEY1 signaling pathway in pulmonary arterial smooth muscle cells reduces pulmonary arterial pressure [50,51].	Arterial EC
LTBP4	Latent transforming growth factor beta binding protein	LTBP4 encodes a protein that binds to transforming growth factor beta (TGFB) while it is being secreted and targeted to the extracellular matrix; defects can cause pulmonary abnormalities such as alveolar septation defects and emphysematous changes in lungs [40].	Arterial EC
FBLN5	Fibulin 5	FBLN5 is involved in the promotion of endothelial cell adhesion through the interaction of integrins and the Arg-Gly-Asp motif [40].	Arterial EC
GJA4	Gap junction protein alpha 4	GJA4 encodes a protein involved in gap junction. Mutations in GJA4 are associated with atherosclerosis and myocardial infarction [40].	Arterial EC
RGCC	Regulator of cell cycle	RGCC is involved in the regulation of cell cycle progression [40].	Capillary EC
SPARC	Secreted protein acidic and cysteine rich	SPARC is involved in extracellular matrix synthesis. SPARC silencing has been shown to reduce proliferation [52].	Capillary EC
SGK1	Serum/glucocorticoid regulated kinase 1	SGK1 contributes to the cellular response to stress by activating potassium, sodium, and chloride channels [40].	Capillary EC
CA4	Carbonic anhydrase 4	This gene encodes a protein member of the zinc metalloenzymes family that catalyzes the reversible hydration of carbon dioxide. The inhibition of carbonic anhydrases has been shown to improve pulmonary inflammation and experimental PH [40,53].	Capillary EC
NR2F2	Nuclear receptor subfamily 2 group F member 2	NR2F2 encodes for a protein member of the steroid thyroid hormone superfamily; plays a role in activating cell cycle genes [40].	Venous EC
ACKR1	Atypical chemokine receptor 1	This gene encodes a glycosylated membrane protein/chemokine receptor that binds and traffics chemokine ligands. ACKR1 is a receptor for chemokines involved in angiogenesis, metastasis, chemotaxis, and cellular retention signals [40,54].	Venous EC
SELP	Selectin P	SELP encodes a P-selectin protein that resides in the Weibel–Palade bodies in endothelial cells. The inhibition or deletion of P-selectin has therapeutic potential and has been shown to reverse pulmonary vascular remodeling and improve right ventricular function in mice [40,55].	Venous EC
PROX1	Prospero homeobox1	PROX1 encodes a protein that is a part of the homeobox transcription factor family and plays a role in embryonic lymphatic development [40].	Lymphatic EC
LYVE1	Lymphatic vessel endothelial hyaluronan receptor 1	LYVE1 encodes type I integral membrane glycoprotein that binds to soluble and immobilized hyaluronan [40].	Lymphatic EC
FLT4	FMS related receptor tyrosine kinase 4	FLT4 encodes tyrosine kinase receptor for vascular endothelial growth factors C and D [40].	Lymphatic EC
PDPN	Podoplanin	PDPN encodes a type-1 integral membrane glycoprotein that is distributed in various human tissues and is a proposed marker of lung injury that plays a role in the development of the heart, lungs, and lymphatic system [40].	Lymphatic EC

**Table 2.** Pulmonary vascular smooth muscle molecular markers and cell adhesion molecules. Smooth muscle cells are the main effectors of vasoconstriction and vasorelaxation which are essential for blood flow and proper vascular tone. Biomarkers frequently used to identify pulmonary vascular smooth muscle cells in research settings are listed below.

Name	Description
α-smooth muscle actin	Plays a role in cell motility, structure, and integrity
Myosin heavy chain 11	Predicted to act upstream of or within smooth muscle contraction
SM22α	Encoded protein is structurally similar to calponin, an actin-binding protein
SM Calponin	Predicted to be involved in negative regulation of vascular associated smooth muscle cell proliferation
H-caldesmon	Enables actin binding activity. Involved in actin filament bundle assembly and positive regulation of protein binding activity
Smoothelin	Structural protein found exclusively in contractile smooth muscle cells
Telokin	Stabilizes unphosphorylated myosin filaments in smooth muscle cells
Metavinculin	Encodes cytoskeletal protein associated with cell–cell and cell–matrix junctions

## Data Availability

No new data were created or analyzed in this study. Data sharing is not applicable to this article.
